# Nutritional Interventions during Chemotherapy for Pancreatic Cancer: A Systematic Review of Prospective Studies

**DOI:** 10.3390/nu15030727

**Published:** 2023-02-01

**Authors:** Marco Cintoni, Futura Grassi, Marta Palombaro, Emanuele Rinninella, Gabriele Pulcini, Agnese Di Donato, Lisa Salvatore, Giuseppe Quero, Giampaolo Tortora, Sergio Alfieri, Antonio Gasbarrini, Maria Cristina Mele

**Affiliations:** 1 UOC di Nutrizione Clinica, Dipartimento di Scienze Mediche e Chirurgiche, Fondazione Policlinico Universitario A. Gemelli IRCCS, Largo A. Gemelli 8, 00168 Rome, Italy; 2 Dipartimento di Medicina e Chirurgia Traslazionale, Università Cattolica del Sacro Cuore, 00168 Rome, Italy; 3 Comprehensive Cancer Center, Dipartimento di Scienze Mediche e Chirurgiche, Fondazione Policlinico Universitario A. Gemelli IRCCS, Largo A. Gemelli 8, 00168 Rome, Italy; 4 Digestive Surgery Unit, Dipartimento di Scienze Mediche e Chirurgiche, Fondazione Policlinico Universitario A. Gemelli IRCCS, Largo A. Gemelli 8, 00168 Rome, Italy; 5 UOC Medicina Interna e Gastroenterologia, Dipartimento di Scienze Mediche e Chirurgiche, Fondazione Policlinico Universitario A. Gemelli IRCCS, Largo A. Gemelli 8, 00168 Rome, Italy

**Keywords:** pancreatic cancer, nutritional support, Oral Nutritional Supplements, body composition, supportive care, Quality of Life

## Abstract

Background: Pancreatic cancer incidence is growing, but the prognosis for survival is still poor. Patients with pancreatic cancer often suffer from malnutrition and sarcopenia, two clinical conditions that negatively impact oncological clinical outcomes. The aim of this systematic review was to analyze the impact of different nutritional interventions on clinical outcomes in patients with pancreatic cancer during chemotherapy. Methods: A systematic review of MedLine, EMBASE, and Web of Science was carried out in December 2022, identifying 5704 articles. Titles and abstracts of all records were screened for eligibility based on inclusion criteria, and nine articles were included. Results: All nine articles included were prospective studies, but a meta-analysis could not be performed due to heterogenicity in nutritional intervention. This Systematic Review shows an improvement in Quality of Life, nutritional status, body composition, oral intake, and Karnofsky Performance Status, following nutritional interventions. Conclusions: This Systematic Review in pancreatic cancer patients during chemotherapies does not allow one to draw firm conclusions. However, nutritional support in pancreatic cancer patients is advisable to ameliorate oncological care. Further well-designed prospective studies are needed to identify nutritional support’s real impact and to establish a reliable way to improve nutritional status of pancreatic cancer patients during chemotherapy.

## 1. Introduction

In 2020, Pancreatic Cancer (PC), with 495,773 new cases, was the 12th most common tumor worldwide and the seventh leading cause of cancer mortality [1]. Incidence is higher in industrialized countries compared to developing countries, suggesting that environmental factors play a significant role as risk factors for the disease [2]. Cigarette smoking, alcohol drinking, physical inactivity, obesity, hypertension, chronic pancreatitis, diabetes, and high cholesterol are recognized as modifiable risk factors for PC development [2,3]. Other risk factors include age, gender, ethnicity, and inherited genetic syndromes [3].

The prognosis of PC patients is generally poor with a relative 5-year survival rate of 10.8%, because it is difficult to diagnose the disease at an early stage, since only 11% of PC are at a local stage at the time of diagnosis, and only a few patients can benefit from surgical resection [4,5]. However, recent progress in diagnosis and chemotherapies give hope for better outcomes in PC patients, even though chemotherapy (CHT) is often burdened with important toxicity, and only fit patients can fully complete the planned treatments [6].

Malnutrition is a common feature in cancer patients due to both cancer itself and the related treatments and, when the neoplasm involves the gastrointestinal system, the maintenance of a proper nutritional balance can be very challenging [7,8]. When the pancreas is the site of cancer, both its exocrine and endocrine functions can be impaired [9]. The altered secretion of pancreatic enzymes determines a series of gastrointestinal symptoms with abdominal pain, bloating, gastric emptying delay, diarrhea, poor appetite, nausea, dyspepsia, malabsorption, and, consequently, weight loss [8,10]. Malnutrition’s prevalence varies between 33.7% and 70.6% in PC patients, while the presence of sarcopenia has a great impact on this population, reaching 74% of PC patients according to some studies [8,11]. Malnutrition and sarcopenia are associated with an increased risk of chemotherapy-related toxicity (CIT), postoperative morbidity, poorer survival, and reduced Quality of Life (QoL) [12,13,14]. Thus, nutritional support during CHT may play a very special role in these patients [15,16].

Therefore, the aim of this systematic review was to analyze the impact of different nutritional interventions on clinical outcomes in PC patients during CHT.

## 2. Materials and Methods

This systematic review was performed according to the Cochrane Handbook for systematic reviews and to the Preferred Reporting Items for Systematic Reviews and Meta-Analyses (PRISMA) statement [17,18] It was registered in the International Prospective Register of Systematic Reviews PROSPERO 2020 CRD42020185706 [19]. The PRISMA checklist is detailed in Table S1.

### 2.1. Eligibility Criteria

We included studies with all the following PICOS Criteria:

- Population: eligible patients must (i) be at least 18 years old with any nutritional status (well-nourished, at risk of malnutrition, and malnourished), (ii) have a PC diagnosis, while (iii) undergoing CHT. Due to the limited number of studies which involve PC patients only, we decided to consider also papers with PC and other gastrointestinal tumors;
- Intervention: studies with nutritional interventions including nutritional counseling, supplementary food or drink, fortified foods, oral nutrition supplements, and enteral or parenteral nutrition during CHT were considered for inclusion in this review;
- Comparison: any types of comparison were considered as possible (i.e., no nutritional intervention, isocaloric diet without specific nutrients, etc.);
- Outcomes: the outcomes considered were CIT, changes in body composition, QoL, survival, and patient’s functional capacity;
- Study designs: eligible study designs included randomized clinical trials (RCTs), prospective non-randomized studies, and other types of prospective studies.

### 2.2. Electronic Searches

The search was carried out on 2 December 2022 using three different electronic databases: Medical Literature Analysis and Retrieval System Online (MedLine) via PubMed, ISI Web of Science (WOS), and Excerpta Medica Database (EMBASE). Databases were screened for search terms in titles and abstract, limiting the search to English papers, without any restriction for date of publication. The comprehensive string search for each database is shown in Table S2.

### 2.3. Study Selection

The study selection process was independently carried out by three reviewers (M.C.; F.G.; M.P.). All articles generated from the electronic search were imported into Mendeley© (Elsevier, Amsterdam, The Netherlands), a reference management software, and duplicates were removed. Titles and abstracts of all records were screened for eligibility based on inclusion criteria, and all judged as ineligible were excluded. After the first title and abstract screening process, the three reviewers performed a second deeper title and/or abstract screening. A full text screening was performed on 43 studies, and 34 were excluded: 14 studies were not prospective ones, 17 were not performed during CHT, and 3 studies had no full text available.

Differences in judgment during the selection process between the three reviewers were settled by discussion and consensus.

### 2.4. Data Extraction

Information was collected using an Excel© (Microsoft Office, Redmond, WA, USA) spreadsheet specifically developed for this study. Each full-text article was retrieved, the ineligible articles were excluded, and the reasoning reported. Differences in judgment during the selection process between the three reviewers were settled by discussion and consensus.

### 2.5. Risk of Bias and Quality Assessment

The risk of bias instruments was used for randomized controlled trials and non-randomized comparative prospective studies. Risk of bias was independently assessed by two reviewers (M.C. and F.G.) and was further entered into the software «Review Manager 5.3.5» (The Nordic Cochrane Centre, Copenhagen, Denmark). According to the “Cochrane Handbook for Systematic Reviews of Interventions” [20], all the articles included were assessed as high, low, or unclear risk of bias.

In total, seven areas were assessed: (1) Random sequence generation; (2) Allocation concealment; (3) Blinding of participants and personnel; (4) Blinding of outcome assessment; (5) Incomplete outcome data; (6) Selective reporting; (7) Other bias (other any important concerns about bias not covered in the other domains (i.e., presence of data regarding diet during other nutritional treatment).

### 2.6. Data Synthesis

Given the high heterogeneity of the studies’ measures, the variability of nutritional intervention, and the variety of the outcomes considered, a meta-analysis resulted unfeasible, and thus, a systematic review was performed. The main results of the review were displayed on a summary of findings table. For each study, a description of the population, type of intervention, outcome measures, and results were presented.

## 3. Results

### 3.1. Study Selection

**Figure 1:**
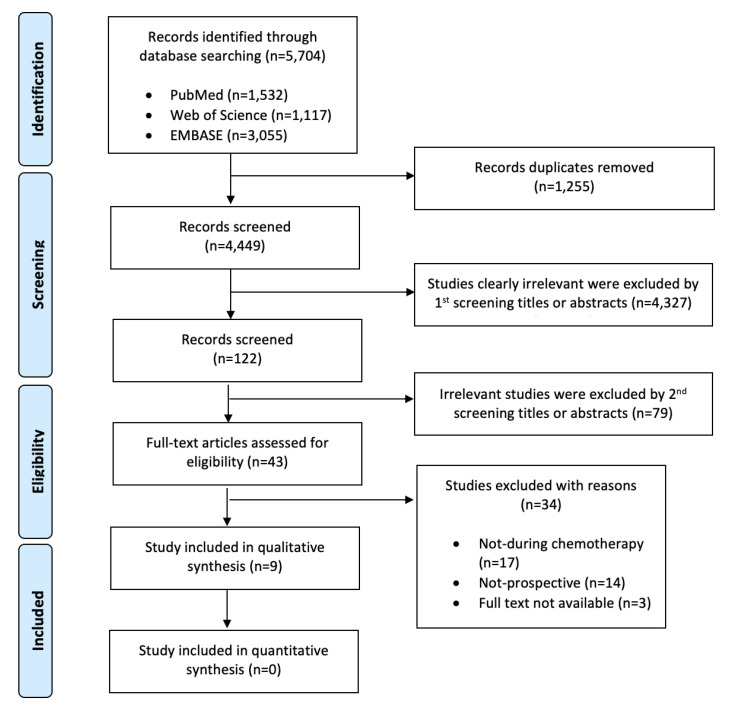
PRISMA Flow Diagram.

The study selection process and the results of the literature search are shown in Figure 1. In particular, starting from the 5704 studies identified from the three different databases (1532 from PubMed, 1117 from Web of Science, and 3055 from EMBASE), nine were finally included into the systematic review process [21,22,23,24,25,26,27,28,29].

### 3.2. Study Characteristics

The most important characteristics of the nine studies are shown in Table 1.

The percentage of PC patients in all the included studies ranges from 7% [25] to 100%, with 6 studies enrolling only PC patients [22,23,24,26,27,29]. The Sample size varies from 7 [21] to 201 patients [25]. Three studies had no comparison groups [21,23,24], two studies had a placebo-controlled group [22,29], and three studies had normal or isocaloric diet as the controlled group [25,27,28]. Four papers considered the use of an Oral Nutritional Supplement (ONS) as nutritional intervention [21,25,27,28], two papers analyzed the role of a parenteral supplementation of fatty acids [23,24], one paper used oral carnitine supplementation [22], and the last one used oral supplement of sulforaphane and glucoraphanin or methylcellulose [29].

Table 2 shows the main characteristics of used ONS. In particular, three papers considered the use of a liquid premixed product [21,27,28], while one used a powder to be mixed with water prior to use [25]. ONS energy intake ranged from 310 [21] to 691 kcal per day [25], while protein intake was from 16 [21] to 45.75 g per day [25].

### 3.3. Study Quality Assessment

The risk of bias was assessed in each included study.

**Figure 2:**
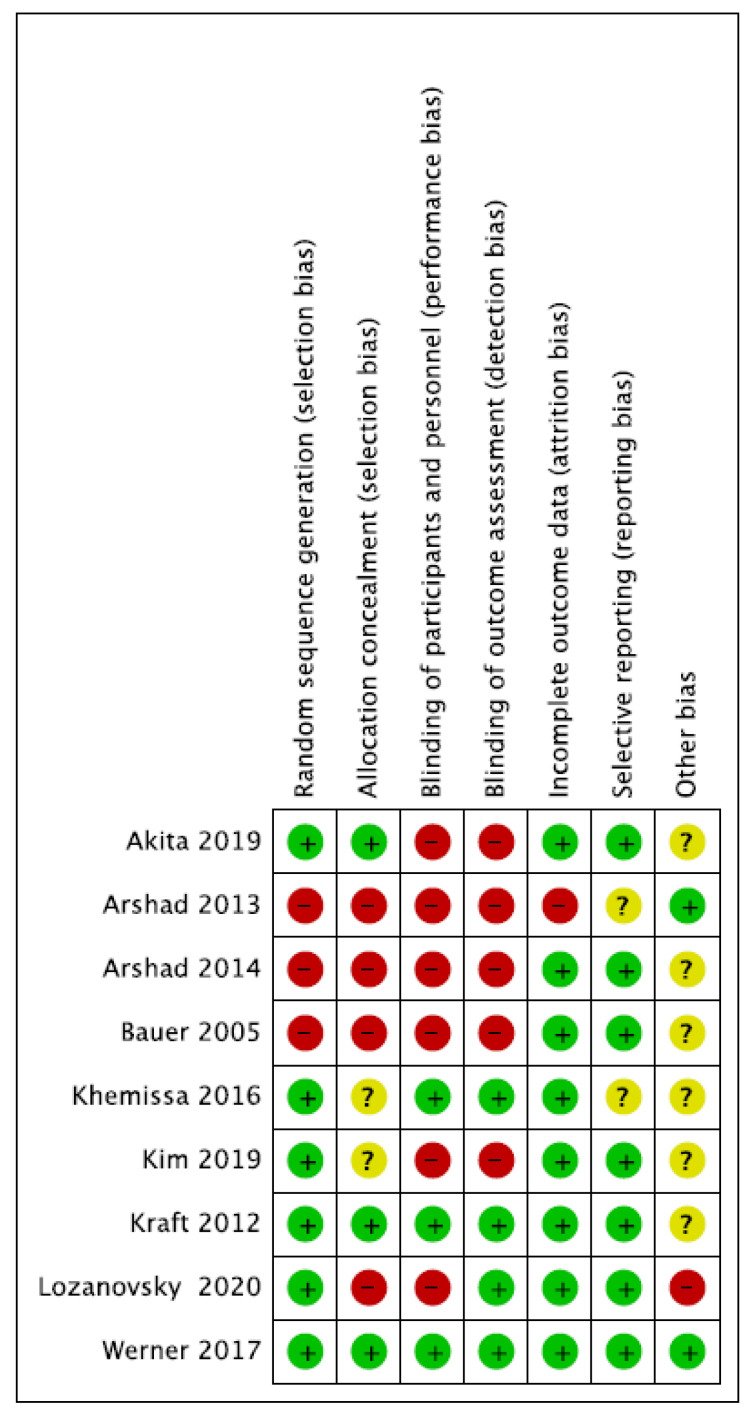
Risk of bias summary [21,22,23,24,25,26,27,28,29].

**Figure 3:**
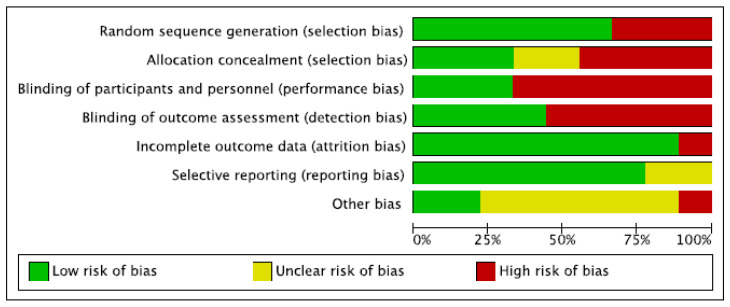
Risk of bias graph.

Figure 2 reports the different types of bias for each study, while Figure 3 shows the cumulative risk of bias expressed in percentage.

### 3.4. Summary of Results

#### 3.4.1. Survival Analysis

Three papers analyzed the impact of nutritional support on survival in PC patients [22,23,29]. In particular, Kraft et al., using oral L-carnitine vs. placebo for 12 weeks, found only a non-statistically significative trend of increased overall survival (OS) (median 519 ± 50 vs. 399 ± 43 days) [22]. Lozanovski et al. showed longer survival in the intervention group (IG) during the first three months after the study (death raw rate of 25% in IG vs. 45% in control group (CG) [29]. Arshad et al. dosed the plasma cytokines at baseline and found a significant correlation between high expression of IL-6 and IL-8 and shorter OS [23]. Moreover, authors evidenced that platelet-derived growth factor (PDGF) and fibroblast growth factor (FGF) serum concentrations decreased at the end of the treatment period and FGF responders had a significantly improved progression free survival (PFS). In the case of PDGF reduction, a tendency toward improved OS was noticed [23].

#### 3.4.2. Quality of Life

Four papers examined the impact of nutritional interventions on QoL [21,22,26,28]. All papers analyzed QoL using the European Organization for Research and Treatment of Cancer (EORTC) QLQ-C30 [21,22,26,28]; only two papers also added the PAN-26 analysis, the QLQ specific for PC [22,26].

In particular, Bauer et al. showed a stability in QLQ-C30 global scale at 4 weeks, with an increase at 8 weeks [21]. Kraft et al. reported an improvement in cognitive functions and global health status, a reduction in gastrointestinal symptoms, while a non-significative difference in fatigue was found [22]. Werner et al. did not find any differences in terms of QoL after 6 weeks of treatment, but only a non-significant slight increase in sub-scale physical, role, social, pain, appetite loss, and global health; moreover, the authors described a significant decrease in hepatic sub-scale of PAN-26 [26]. The study by Kim et al. reported a non-statistical increase in QoL in both IG and CG, while a decrease in subscale fatigue in IG and a pain reduction in CG was described [28].

#### 3.4.3. Chemotherapy-Induced Toxicity

CIT was observed in two studies [25,27]. In the phase III study from Khemissa et al., the authors aimed to evaluate the possible role of oral supplementation with glutamine and TGF-β2 in the prevention of grade 3 and 4 non-hematological CIT. However, the results did not confirm this hypothesis, and no difference was evidenced between IG and CG for all kinds of CITs [25]. Akita et al. analyzed the incidence of adverse events during neoadjuvant CHT between patients on a normal diet and those who received hypercaloric, Eicosapentaenoic Acid (EPA)-enriched oral supplements; even in this case, no significant difference between the two groups in terms of CIT was evidenced [27].

#### 3.4.4. Nutritional Status

Only two studies evaluated PC patients’ nutritional status with a Patient-Generated Subjective Global Assessment (PG-SGA) score [21,28]. According to this score, patients could be defined: well nourished (PG-SGA A), moderated or suspected of being malnourished (PG-SGA B), and severely malnourished (PG-SGA C). Bauer et al. showed a significant reduction in PG-SGA score from the baseline to 8 weeks (median 13 range 4.0–19.0 vs. median 4 range 1.0–16.0, *p* = 0.019); this improvement was significantly associated with a change in QoL (*p* = 0.020), Karnofsky Performance Status (KPS) (*p* = 0.009), and lean body mass (*p* = 0.040) [21]. Kim et al. revealed a reduction on PG-SGA score after 8 weeks of intervention (9.5 ± 0.9 vs. 5.6 ± 0.8, *p* = 0.002) [28].

#### 3.4.5. Body Composition

Five studies evaluated the effect of different nutritional interventions on patients’ body weight and/or body composition [21,22,26,27,28].

Akita et al. showed a higher skeletal muscle mass in those patients who consumed more than 50% of the prescribed ONS (*p* 0.042) and an improvement in psoas muscle ratio in the same population [27]. Another study evaluated the effect of n-3 fatty acid-enriched ONS reporting a clinical improvement in weight and lean body mass although not statistically significant [21]. Werner et al. showed a significant body weight stabilization, with a gain of body weight in half of the patients, but no significative change in fat mass, muscle mass, or body water was detected [26]. In addition, Kim et al. evaluated the effect of ONS administration in PC patients showing a significantly increase of fat mass from the baseline to 8 weeks of intervention, and a stabilization of fat-free mass, skeletal muscle mass, and body cell mass [28]. In the study by Kraft et al., who evaluated the effect of carnitine supplementation, an increase of body weight in the IG, as well as increase in body cell mass and body fat mass, was found [22].

#### 3.4.6. Oral Intake

Three studies evaluated the effect of nutritional intervention on oral intake in PC patients undergoing CHT [21,26,28]. Bauer et al. showed no reduction of meal protein and energy intake with supplementation and observed 1.4 (1.2–2.2) g/kg/day of total protein intake and 33 (25–42) kcal/kg/day of total energy intake after 8 weeks of intervention [21]. Kim et al. revealed significant increases in dietary intakes of calories (1488.1 kcal vs. 1946.4 kcal, *p* = 0.001), proteins (64.1 g vs. 89.9 g, *p* = 0.001), carbohydrates (247.9 g vs. 289.2 g, *p* = 0.015), and lipids (38.6 g vs. 51.9 g, *p* = 0.023) in the ONS group from the baseline to 8 weeks of intervention. However, there was no significant difference between the change of values of dietary intake between baseline and 8 weeks in the ONS and non-ONS group [28]. Werner et al. evaluated appetite and meal portions in PC patients supplemented with n-3 fatty acids from MPL or FO and showed stabilization of appetite in both groups of patients. Moreover, meal portions increased significantly in the FO group (*p* = 0.02) and MPL group (*p* = 0.05) [26].

#### 3.4.7. Karnofsky Performance Status

Two papers considered variation in patients’ functional capacity, measured according to KPS [21,29]. Bauer et al. showed that nutritional intervention with high-protein, high-calorie nutritional supplement containing EPA, not only improved patients’ nutritional status but equally increased their KPS after 8 weeks of treatment (*p* = 0.01) [21]. Similarly, Lozanovski et al. described a decrease in KPS in both groups (intervention and control group), stating that broccoli sprouts did not impact patient self-care and overall abilities severely [29].

## 4. Discussion

At our knowledge, this is the first Systematic Review on nutritional intervention in PC patients, during chemotherapies, enrolling only prospective studies. Even though nutritional interventions in PC patients should be a routine [7], the number of robust studies remains scarce.

Nutritional intervention strategy should include personalized nutritional counseling with a trained physician or dietitian specialized in oncological cures [30], with the evaluation of nutritional targets and intakes. According to the European Society of Nutrition and Metabolism (ESPEN) guidelines on nutrition in cancer patients, an energy intake of 25–30 kcal/kg/day and a protein intake of 1.0–1.5 g/kg/day should be guaranteed to all cancer patients [7,31,32]. However, only a few papers analyzed PC patients’ nutritional requirements and performed a malnutrition risk assessment. To modify this situation, an integration in the cure pathway to include nutritional evaluation and intervention in clinical routine for oncological patients was proposed [33].

QoL represents a major concern in PC patients undergoing CHT treatments [34]. In addition to the more generic QLQ-C30 questionnaire, valid for all oncological patients, a specific module called PAN26 has been developed for the evaluation of the QoL in PC patient; however, QoL was analyzed in only four studies (less than half), and the specific module PAN26 was studied in only two of them. While data from the literature showed a general improvement in QoL in oncological patients who undergo nutritional interventions [35,36], our results are not conclusive; in fact, two papers showed an increase in QoL, one paper showed an increase only in subscale fatigue, while one other showed no differences.

KPS is a scale that tries to quantify the patient’s well-being and their capacity to do all the daily-life activities [37]. In the two papers enrolled in this Systematic Review, nutritional supplementation showed an increase in patient function. In line with this, the same results were obtained also in other neoplasm-affected patients [38,39], demonstrating that better nutritional status is related to better functional capacity and physical resistance to therapies.

Different papers suggest an association between body composition and treatment related toxicity. In particular, sarcopenia is related with an increased incidence of severe adverse reactions and treatment interruption [40,41,42,43]. In patients with pancreatic neoplasm, results are still not conclusive [44]. In our systematic review, only two studies analyzed the effect of nutritional intervention on CIT, but no significant correlation was found [25,27].

Notably, malnutrition affects prognosis and survival in PC patients [45,46,47]. However, studies reporting the effects of high-energy ONS on survival outcomes are limited and heterogeneous, and there is no consensus [48,49,50]. None of the papers included in our review that supplemented PC patients with ONS evaluated their effect on survival. In our analysis, one study reported longer survival after three months with daily supplementation of broccoli sprouts [29]. Moreover, a trend of increase in OS was found with oral supplementation of L-carnitine (4 g/day) for 12 weeks, even if no statistical significance was achieved [22].

Recently a prospective cohort study showed that daily protein intake influenced the prognosis of patients with unresectable PC undergoing CHT. Interestingly, authors found that protein intake <1.1 g/kg/day was an independent poor prognostic factor in this setting [49]. Since the loss of appetite and the consequent reduction of calories and protein intake are common features of PC patients, the use of ONS can be an advisable, powerful strategy. Papers included in our analysis globally demonstrated an increase of dietary intake of all macronutrients with the use of ONS [21,26]. Oral supplementation with n3-fatty acids induced a stabilization of appetite and meal portions tended to increase [28].

Furthermore, the use of ONS seemed to have a direct impact on malnutrition. Indeed, two studies reported a significant reduction in PG-SGA score after 8 weeks of intervention with high-energy and high-protein oral supplementation [21,28].

Globally, studies focusing on nutritional interventions in gastrointestinal (GI) cancer showed heterogeneous results. A systematic review conducted on GI cancers (stomach, esophagus, pancreas) undergoing surgery presented scarce evidence of the effectiveness of using ONS in terms of body weight gain and increased energy intake both in pre- and post-operative period [51]. However, a meta-analysis based only on gastric cancer patients highlighted a positive association between the use of ONS and reduced weight loss, especially in the postoperative period [50]. Another recent meta-analysis on the role of oral supplementation with an amino acid-enriched formula containing glutamine, vitamins, and minerals during CHT and/or radiotherapy in 445 patients with GI and head-neck cancer showed that this type of nutritional intervention could be beneficial in preventing CIT and, in particular, oral mucositis [52]. In other malignancies, a proper nutritional intervention is associated with benefits in terms of body weight and body composition [53,54,55]. However, data on the correct timing and the proper type of nutritional intervention are still unconclusive. Results from our study are in line with these findings [21,22,26,27,28].

Moreover, in PC, particular attention should be paid to pancreatic exocrine insufficiency (PEI). Notably, the reduction of pancreatic secretions leads to maldigestion and malabsorption and remarkably contributes to the development of malnutrition. Thus, when considering nutritional intervention in PC, pancreatic enzyme replacement therapy (PERT) must always be taken into account. PEI can be caused by local tumor-induced changes (i.e., Warburg effect, production of tumor-specific factors, tumor location, etc.) or can be the consequence of surgery [56]. It can be present even before the onset of clinical symptoms, and the estimated prevalence in patients with advanced PC is 72% [57,58]. A few studies evidence a positive association between PERT prescription with survival and QoL [59,60]. However, PERT is not always adequate in common practice and frequently enzyme dosages are lower than needed [58]. According to this observation, none of the studies collected in our review considered PERT. Due to the complex etiology of malnutrition in PC patients, we believe that close attention should be given to any aspect that can improve nutritional status and that PERT must be part of nutritional intervention.

The present Systematic Review has some limitations: (i) the small number of included studies (only nine papers); (ii) the necessity to include papers which enrolled PC patients during CHT together with other gastrointestinal cancers; (iii) the large variability in term of nutritional intervention, population, and outcomes.

## 5. Conclusions

Pancreatic cancer remains one of the most challenging cancers for oncologists and surgeons. Due to the paucity of studies, the scarcity of sample size, the heterogeneity of the studies, and the lack of robust randomized clinical trials, it is not feasible to draw strong conclusions on the role of nutritional support during CHT for PC patients. The main results of this Systematic Review are an improvement in QoL, nutritional status, body composition, oral intake, and KPS when nutritional support is provided in PC patients. Nonetheless, nutritional intervention in PC patients remains advisable, particularly during CHT, to contribute to the oncological care.

Nevertheless, further well-designed prospective studies are needed to identify the real impact of nutritional support during oncological pathway in PC patients and to establish the most effective strategy aiming to reduce the burden of malnutrition in this population.

## Figures and Tables

**Table 1:** Main characteristics of included studies.

Refs	Author and Year	Study Design	% Pancreatic Cancer	Sample Size (IG/CG)	Time of Intervention	Type of Nutritional Intervention	Comparison	Results
[21]	Bauer JD 2005	Single-arm trial	71.4	7 (7/0)	8 weeks	ONS	-	- ↑ protein (*p* = 0.011), energy (*p* = 0.011), and fiber (*p* = 0.006) intake ↑ nutritional status (*p* = 0.019) ↑ KPS (*p* = 0.01) ↑ QoL(*p* = 0.019) Not statistically significant improvements in: - BW (*p* = 0.368) - LBM (*p* = 0.225)
[22]	Kraft M et al. 2012	Prospective, multi-center, placebo-controlled, randomized, and double-blinded trial	100	72 (38/34)	12 weeks	Oral liquid formulation of L-Carnitine	Placebo	in the IG group vs. CG: ↑ BCM after 6 weeks (*p* = 0.013) ↑ BF after 12 weeks (*p* = 0.041) ↑ BMI after 12 weeks (*p* < 0.018) ↑ cognitive function after 6 weeks (*p* < 0.034) ↑ global health status after 12 weeks (*p* < 0.041) ↓ gastrointestinal symptoms after 12 weeks (*p* < 0.033) No significant differences between the two groups in survival
[23]	Arshad A et al. 2013	Single-arm phase II clinical trial	100	32 (32/0)	Weekly for 3 weeks followed by a rest week during the CHT period	Parenteral supplement n-3FA-rich lipid emulsion	-	↓ OS in high expressors of IL-6 (*p* = 0.009) and IL-8 (*p* = 0.02) ↓ PFS in high expressors of IL-8 (*p* = 0.002)
[24]	Arshad A et al. 2014	Single-arm phase II clinical trial	100	21 (21/0)	Weekly for 3 weeks followed by a rest week for up to six months	Parenteral supplement n-3FA-rich lipid emulsion	-	Over the entire treatment course of up to six months: ↑ ECM pellet uptake of EPA (*p* = 0.005) and DHA (*p* < 0.001) ↓ n6:n3 ratio (*p* < 0.001)
[25]	Khemissa F et al. 2016	Double-blind, randomized, controlled, and multicenter trial	7	201 (99/102)	Five days before the start of each CHT cycle	ONS	Isocaloric ONS	No significant differences between the two groups in term of compliance and toxicities
[26]	Werner K et al. 2017	Randomized, double-blind, controlled trial	100	60 (31/29)	6 weeks	FO capsules	MPL capsules	in both groups: BW stabilization (*p* = 0.001 in FO group; *p* = 0.003 in MPL group) ↑ meal portions (*p* = 0.02 in FO group; *p* = 0.05 in MPL group) No significant changes in both groups in QoL, and food intake.
[27]	Akita H et al. 2019	RCT	100	62 (31/31)	5 weeks	ONS	Normal diet	in CG group: ↓ Post/pre ratio of SMM (*p* = 0.014) in both groups: ↓ PMA (IG *p* = 0.002; CG *p* < 0.001) ↓ BMI (IG *p* = 0.011; CG *p* = 0.001) in IG group: ↑ Post/pre ratio of PMA (*p* = 0.001) ↑ Post/pre ratio of SMM (*p* = 0.042) ↑ Post/pre ratio of PMA (*p* < 0.001) No significant difference between the two groups in NACRT-related toxicity
[28]	Kim SH et al. 2019	Prospective randomized study	29.4	58 enrolled (36/22)	8 weeks	ONS	Nutritional care only	No significant difference between the two groups in BW, FFM, SMM, BCM, QoL, and biochemical tests (all patients) (dividing population based on CHT cycles) In IG vs. CG: ↑ dietary intake ↓ reduction of fatigue (*p* = 0.041) ↑ PG-SGA grade ratio (*p* < 0.05) ↑ BW (*p* = 0.049) ↑ FFM (*p* = 0.034) ↑ SMM (*p* = 0.049) ↑ BCM (*p* = 0.049)
[29]	Lozanovski et al. 2020	Prospective, placebo-controlled trial	100	40 (29/11)	12 months	Daily intake of broccoli sprouts containing 90 mg sulforaphane and 180 mg glucoraphanin or methylcellulose a	Placebo	In IG: Drop out: 72% IG vs. CG: ↑ Survival at 180 days (*p* = 0.291)

BCM: Body Cell Mass; BF: Body Fat; BMI: Body Mass index; BW: Body Weight; CAF: pro-angiogenic cytokines and growth factors; CG: control group; DHA: Docosahexaenoic Acis; ECM: Erythrocyte Cell Membrane; EPA: Eicosapentaenoic Acid, FFM: Fat-Free Mass; FO: fish oil; KPS: Karnofsky Performance Status; IG: intervention Group; LBM: Lean Body Mass; NACRT: Neoadjuvant Chemoradiotherapy; MPL: Marine Phospholipids; OS: Overall Survival; PFS: Progression Free Survival; PG-SGA: Patient-Generated Subjective Global Assessment; PMA: Psoas Major Muscle Area; QoL: Quality of Life; SMM: Skeletal muscle mass.

**Table 2:** Main characteristics of Oral Nutritional Supplements used in the enrolled studies.

Refs	Author	ONS Type	ONS Quantity	Amount (per Day)	Energy (kcal per Day)	Protein (g per Day)	Other
[21]	Bauer JD et al.	L	Not reported	At least 1	310	16	1.1 g EPA
[25]	Khemissa F et al.	P	75 g	2	691	45.75	13.5 g glutamine + TGF-β2 20 mg
[27]	Akita H et al.	L	220 mL	2	560	29.3	1.98 g EPA
[28]	Kim SH et al.	L	150 mL	2	400	18	2.5 g fiber

Abbreviations: EPA: Eicosapentaenoic Acid; L: Liquid Formula; ONS: Oral Nutritional Supplements; P: Powder Formula; Refs: Bibliographic references; TGF-β2: Tumor Growth Factor.

## Data Availability

No new data were created or analyzed in this study. Data sharing is not applicable to this article.

## Supplementary Materials

The following supporting information can be downloaded at: https://www.mdpi.com/article/10.3390/nu15030727/s1; Table S1: PRISMA Checklist; Table S2: Full search strategies for electronic databases.
