# Supplementary material for: Nutritional Interventions during Chemotherapy for Pancreatic Cancer: A Systematic Review of Prospective Studies
Source: Nutrients. 2023 Feb 1;15(3):727. doi: 10.3390/nu15030727 (PMC9920549; doi:10.3390/nu15030727)
Supplement: Supplementary file 1 [file nutrients-15-00727-s001.zip › Table S2.pdf]

Table S2: Full search strategies for electronic databases.

**Database**	**PUBMED**	
**Date**	**02 12 22**	
**Search strategy**		**Results**
**#1**	diet*[TextWord] OR diet therapy[MeSHTerms] OR therapy nutrition[MeSHTerms] OR eat*[TextWord] OR food*[TextWord] OR feed*[TextWord] OR meal*[TextWord] OR nutriment*[TextWord] or nutritional advice*[TextWord] OR nutritional counseling*[TextWord] OR nutritional support*[TextWord] OR nutritional intervention*[TextWord]	2,642,584
**#2**	cancer*[TextWord] OR oncology [TextWord] OR tumour*[TextWord] OR tumor*[TextWord] OR malignan*[TextWord] OR carcinoma [TextWord] OR neoplasm*[TextWord]	5,535,398
**#3**	pancreas [MeSHTerms] OR pancreatic* [TextWord]	366,604
**#4**	#2 AND #3	168,626
**#5**	#1 AND #4	10,512
**#6**	#5 AND prospective study OR cohort study (prospective study OR cohort study OR randomised controlled trial* OR randomised controlled clinical trial* OR randomised controlled study OR randomised controlled clinical study OR randomized controlled trial* OR randomized controlled clinical trial* OR randomized controlled stud* OR randomized controlled clinical stud* OR randomised-controlled trial* OR randomised-controlled clinical trial* OR randomised-controlled study OR randomised-controlled clinical study OR randomized-controlled trial* OR randomized-controlled clinical trial* OR randomized-controlled stud* OR randomized-controlled clinical stud*)	1,615
**#7**	**#6 Refined for HUMAN STUDY**	**1,532**
**Database**	**WEB OF SCIENCE**	
**Date**	**02 12 22**	
**Search strategy**		**Results**
**#1**	(diet* OR eat* OR food* OR feed* OR meal* OR nutriment* or nutritional advice* OR nutritional therapy* OR nutritional support* OR nutritional intervention*)	3,525,476
**#2**	pancreas OR pancreatic	314,759
**#3**	(cancer* OR oncology OR tumour* OR tumor* OR malignan* OR carcinoma OR neoplasm*)	4,395,053

**#4**	(prospective study OR cohort study OR randomised controlled trial* OR randomised controlled clinical trial* OR randomised controlled study OR randomised controlled clinical study OR randomized controlled trial* OR randomized controlled clinical trial* OR randomized controlled stud* OR randomized controlled clinical stud* OR randomised-controlled trial* OR randomised-controlled clinical trial* OR randomised-controlled study OR randomised-controlled clinical study OR randomized-controlled trial* OR randomized-controlled clinical trial* OR randomized-controlled stud* OR randomized-controlled clinical stud*)	1,748,334
**#5**	**#1 AND #2 AND #3 AND #4**	**1,117**
**Database**	**EMBASE**	
**Date**	**02 12 22**	
**Search strategy**		**Results**
**#1**	cancer* OR oncology OR tumour* OR tumor* OR malignan* OR carcinoma OR n eoplasm*	7,265,519
**#2**	pancreas OR pancreatic*	534,464
**#3**	diet* OR eat* OR food* OR feed* OR meal* OR nutriment* OR (nutritional AND advice*) OR (nutritional AND therapy*) OR (nutritional AND support*) OR (nutritional AND intervention*)	3,825,898
**#4**	prospective AND study OR (cohort AND study) OR (randomised AND controlled AND trial*) OR (randomised AND controlled AND clinical AND trial*) OR (randomised AND controlled AND study) OR (randomised AND controlled AND clinical AND study) OR (randomized AND controlled AND trial*) OR (randomized AND controlled AND clinical AND trial*) OR (randomized AND controlled AND stud*) OR (randomized AND controlled AND clinical AND stud*) OR ('randomised controlled' AND trial*) OR ('randomised controlled' AND clinical AND trial*) OR ('randomised controlled' AND study) OR ('randomised controlled' AND clinical AND study) OR ('randomized controlled' AND trial*) OR ('randomized controlled' AND clinical AND trial*) OR ('randomized controlled' AND stud*) OR ('randomized controlled' AND clinical AND stud*)	3,783,679
**#5**	**#1 AND #2 AND #3 AND #4**	**3,055**
